# The influence of beam parameters on FLASH effect

**DOI:** 10.3389/fonc.2025.1431700

**Published:** 2025-04-22

**Authors:** Binwei Lin, Huan Du, Xiaofei Hao, Yuwen Liang, Haonan Xu, Wenqiang Tang, Jie Li, Yu Zhang, Xiao Bo Du

**Affiliations:** ^1^ Department of Oncology, National Health Commission (NHC) Key Laboratory of Nuclear Technology Medical Transformation (Mianyang Central Hospital), Mianyang Central Hospital, School of Medicine, University of Electronic Science and Technology, Mianyang, China; ^2^ Sichuan Clinical Research Center for Radiation and Therapy, Mianyang, Sichuan, China; ^3^ Department of Oncology, Affiliated Hospital of North Sichuan Medical College, Nanchong, China

**Keywords:** ultra-high dose rate radiotherapy, FLASH effect, dose rate, total dose, pulse structure

## Abstract

Ultra-high dose rate radiotherapy (FLASH-RT) is typically defined as an external beam radiotherapy that utilizes a dose rate of 40 Gy/s or higher, compared with conventional dose rate radiotherapy (≤0.1 Gy/s). The primary advantage of FLASH-RT lies in its ability to minimize damage to organs at risk surrounding the cancer while preserving the anti-tumor effect. This phenomenon, known as the FLASH effect, has been widely studied in various bodily systems. However, recent publication of negative research findings related to FLASH-RT warrant a reassessment of whether this definition is accurate. Therefore, this review aims to critically examine how various beam parameters impact the manifestation of the FLASH effect. Following extensive literature review, we propose that an average dose rate of 40 Gy/s to be the lowest dose that triggers the FLASH effect. Beyond this threshold, different organs, including the brain, lungs, intestine, and skin, required varying minimum single total doses to trigger FLASH effects, with a trend of enhanced FLASH-RT protective effects as the single total doses increased. Moreover, single or multiple pulses and the characteristic parameters of the pulse structure, including single pulse dosage, pulse width, pulse interval, pulse frequency, and total irradiation time, were found to also impact the FLASH effect.

## 1 Introduction

Ultra-high dose rate radiotherapy (FLASH-RT) refers to a radiation therapy technique that utilizes ultra-high dose rates (≥40 Gy/s) to treat cancer (1). Compared to conventional dose rate radiotherapy (CONV-RT, ≤0.1 Gy/s), FLASH-RT offers the advantage of reducing damage to organs at risk (OARs) surrounding the cancer while maintaining anti-tumor efficacy (2), in a phenomenon known as the FLASH effect. When tumor dose equivalence is achieved with CONV-RT, FLASH-RT can reduce OAR toxicity and improve treatment safety. Owing to the higher OAR tolerance dose, FLASH-RT can deliver higher doses of radiation to the tumor area, potentially further improving efficacy for radiation-insensitive tumors. Additionally, FLASH-RT can complete treatment within a very short amount of time (<1 min), compared to the weeks required by CONV-RT (3); thus, reducing patient treatment duration and economic burden.

Preclinical studies have demonstrated the protective benefits of FLASH-RT in the brain (4), lungs (2), intestines (5), skin (6), and circulating immune cells (7) without consuming anti-tumor effects (2, 8–11). The first clinical study involving FLASH-RT conducted in 2019 (12) demonstrated complete remission in a patient with skin T-cell lymphoma who received a single total FLASH-RT dose of 15 Gy (electron, 166.7 Gy/s), with only grade 1 skin toxicity reported (12). Recently, the FAST-01 clinical study showed promising results in ten patients with bone metastases in the extremities who received a total FLASH-RT dose of 8 Gy (proton, 51–61 Gy/s), reporting a pain relief rate of 67%, complete relief rate of 50%, and no grade 3 treatment-related toxicities (13). The confirmed safety and effectiveness of FLASH-RT for the treatment of bone metastasis in the FAST-01 study, lead to the initiation of the FAST-02 study (proton FLASH-RT for the treatment of symptomatic bone metastases in the thorax), which began recruiting patients in 2022. Thus, these encouraging results have made FLASH-RT a focus of attention in radiotherapy. However, recent reports of negative results related to FLASH-RT (**Table 1**) indicate that the characterization of FLASH-RT solely based on a dose rate ≥40 Gy/s may be unreasonable. In this review, we consolidate existing research on FLASH-RT beam parameters and discuss their impact on the FLASH effect by focusing on dose rate, total dose, and pulse structure, which are considered crucial factors impacting FLASH effect production. **Table 1** summarizes *in vivo* studies exploring these parameters as variables.

**Table 1:** Summary of *in vivo* studies using beam parameters as variables.

System	*In vitro*/*In vivo*	Author(s)	Year	Model	Radiation type	Total dose/fractions	Mean dose rate (Gy/s)	Pulse width (microsecond)	Dose per pulse,Gy	Frequency, Hz	Instantaneous dose rate (Gy/s)	Protective effect*
Brain	*In vivo*	Montay-Gruel P (14)	2017	Mice	Electrons	10Gy/1fx	1	1.8	0.01	100	5.6× 10^3^	No
							3	1.8	0.03	100	1.7 × 10^4^	No
							10	1.8	0.1	100	5.6 × 10^4^	No
							30	1.8	0.3	100	1.7 × 10^5^	No
							60	1.8	0.6	100	3.5 × 10^5^	Yes
							100	1.8	1	100	5.6 × 10^5^	Yes
							500	1.8	5	100	2.8 × 10^6^	Yes
							5.6 × 10^6^	1.8	10	Single pulse	5.6 × 10^6^	Yes
		Montay-Gruel P (8)	2021	Mice	Electrons	10Gy/1fx	5.6 × 10^6^	1.8	10-14	Single pulse	5.6-7.8 × 10^6^	Yes
						14Gy/1fx	7.8 × 10^6^	1.8	10-14	Single pulse	5.6-7.8 × 10^6^	No
						14Gy/2fx	3.9 × 10^6^	1.8	10-14	Single pulse	5.6-7.8 × 10^6^	Yes
						14Gy/4fx	1.9 × 10^6^	1.8	10-14	Single pulse	5.6-7.8 × 10^6^	No
						30Gy/3fx	5.6 × 10^6^	1.8	10-14	Single pulse	5.6-7.8 × 10^6^	Yes
						25Gy/1fx	2.5 × 10^3^	1.8	10-14	Single pulse	5.6-7.8 × 10^6^	No
	*In vivo*	Montay-Gruel P (15)	2019	Mice	Electrons	10Gy/1fx	>100	1.8	1	100	5.6 × 10^5^	Yes
						12Gy/1fx		1.8	1	100	5.6 × 10^5^	Yes
						14Gy/1fx		1.8	1	100	5.6 × 10^5^	No
		Allen BD (16)	2020	Mice	Electrons	10Gy/1fx	5.6 × 10^6^	1.8	1	100	5.6 × 10^5^	Yes
						25Gy/1fx	2500	1.8	2	100	5.6 × 10^5^	Yes
Intestine	*In vivo*	Ruan JL (17)	2021	Mice	Electrons	7.5~12.5Gy/1fx	2.2~5.9× 10^6^	3.4	7.5-20	Single pulse	2.3-5.9 × 10^6^	Yes
						11.2Gy/12.5 Gy/1fx	≥280	3.4	7.5-20	Single pulse	2.3-5.9 × 10^6^	Yes
						11.2/12.5Gy/1fx	<280	3.4	7.5-20	Single pulse	2.3-5.9 × 10^6^	No
	*In vivo*	Zhu H (18)	2022	Mice	X-rays	10Gy/1fx	>150	NA	NA	Single pulse	NA	Yes
						15Gy/1fx	>150	NA	NA	Single pulse	NA	Yes
	*In vivo*	Zhang Q (19)	2023	Mice	Protons	14Gy/1fx	120	3 × 10^-3^	1.132 × 10^-6^	1.06×10^8^	377	No
						15.1Gy/1fx	120	3 × 10^-3^	1.132 × 10^-6^	1.06×10^8^	377	No
						16Gy/1fx	120	3 × 10^-3^	1.132 × 10^-6^	1.06×10^8^	377	No
						16.2Gy/1fx	120	3 × 10^-3^	1.132 × 10^-6^	1.06×10^8^	377	No
						17Gy/1fx	120	3 × 10^-3^	1.132 × 10^-6^	1.06×10^8^	377	No
						17.5Gy/1fx	120	3 × 10^-3^	1.132 × 10^-6^	1.06×10^8^	377	No
						18Gy/1fx	120	3 × 10^-3^	1.132 × 10^-6^	1.06×10^8^	377	No
	*In vivo*	Levy K (9)	2020	Mice	Electrons	12Gy/1fx	216	5	2	10^8^	4 × 10^5^	Yes
						14Gy/1fx	216	5	2	10^8^	4 × 10^5^	Yes
						16Gy/1fx	216	5	2	10^8^	4 × 10^5^	Yes
Skin	*In vivo*	Soto LA (20)	2020	Mice	Electrons	30/40Gy/1fx	180	5	2	90	4 × 10^5^	Yes
						10/16/20Gy/1fx	180	5	2	90	4 × 10^5^	No
						50Gy/1fx	83	NA	NA	NA	NA	No
	*In vivo*	Velalopoulou A (21)	2021	Mice/ cannie	Protons	30Gy/1fx	69–124	NA	NA	NA	NA	Yes
						45Gy/1fx	69–124	NA	NA	NA	NA	Yes
	*In vivo*	Singers Sørensen B (22)	2021	Mice	Protons	31.2-53.5Gy/1fx	65-92 Mean:80	NA	NA	N	NA	Yes
	*In vivo*	Miles D (23)	2023	Mice	X-rays	35Gy/1fx	87	4 × 10^5^	1	100	NA	Yes
						43Gy/1fx	87	5 × 10^5^	1	100	NA	No
	*In vivo*	Rohrer Bley C (24)	2022	Cat	Electrons	30Gy/1fx	1500	1.8	3	100	5.5-5.9 × 10^6^	No
				Mini Pig		31Gy/1fx	163	1.8	20	100	8.61 × 10^5^	No
	*In vivo*	Gaide O (25)	2021	Human (cutaneous lymphoma)	Electrons	15 Gy/1fx	166	1	1.5	100	1.5 × 10^6^	No
Immune system	In silico study	Jin JY (26)	2020	In silico study	NA	2Gy/1fx	0.0017~ 333	NA	NA	NA	NA	No
						>2~50 Gy/1fx	<40	NA	NA	NA	NA	No
						>2~50 Gy/1fx	≥40	NA	NA	NA	NA	Yes
	*In vitro*	Bozhenko VK (7)	2019	Raji/ Jurkat/ lymphocyte	γ-rays	1Gy/1fx	1.66-6.66 × 10^7^	16.6	NA	NA	NA	Yes
						2Gy/1fx	3.33 × 10^7^	16.6	NA	NA	NA	Yes
						3Gy/1fx	4.98 × 10^7^	16.6	NA	NA	NA	Yes
						4Gy/1fx	6.66 × 10^7^	16.6	NA	NA	NA	Yes
Others	*In vivo*	Beyreuther E (27)	2019	Zebrafish	Protons	>15~40Gy/1fx	100	>10^5^	NA	NA	500	Yes
						≤15 Gy/1fx	100	>10^5^	NA	NA	500	No
	*In vivo*	Karsch L (28)	2023	Zebrafish	Protons	31.9 ± 0.5Gy/1fx	286.7	5× 10^-6^	22	1.3× 10^7^	4.4 × 10^6^	Yes
						32.3 ± 0.6 Gy/1fx	177.2	5× 10^-6^	7.0 × 10^3^	1.3× 10^7^	1.4 × 10^9^	Yes
						32.1 ± 0.6 Gy/1fx	2.5× 10^5^	5× 10^-6^	7.5 × 10^3^	1.3× 10^7^	1.5 × 10^9^	Yes
					Protons	30.1 ± 0.8 Gy/1fx	300	2× 10^-3^	3	NA	1.5 × 10^3^	Yes
lung	*In vivo*	Favaudon V (2)	2014	Mice	Electrons	15GY/1fx	60	1	<5	100-150	10^6^-10^7^	No
						17GY/1fx	60	1	<5	100-150	10^6^-10^7^	YES
						23GY/1fx	60	1	<5	100-150	10^6^-10^7^	Yes
						28Gy/1fx	60	1	<5	100-150	10^6^-10^7^	Yes
	*In vivo/In vitro*	Fouillade C (29)	2019	Cells/ Mice	Electrons	5.2Gy/1fx	60	NA	NA	NA	NA	Yes
						2Gy/1fx	60	NA	NA	NA	NA	Yes
						4Gy/1fx	60	NA	NA	NA	NA	Yes
						17Gy/1fx	60	NA	NA	NA	NA	Yes
Gonadal system	*In vivo*	Cuitiño MC (30)	2023	Mice	Electrons	5Gy/1fx	2.35 × 10^6^ Gy/s	1.8	1	60	2.35 × 10^6^	No
						8Gy/1fx	2.31 × 10^6^ Gy/s	4	1	60	2.31 × 10^6^	No
						16Gy/fx1	234 Gy/s	4	5	60	1 × 10^6^	No
Tumor	*In vivo*	Konradsson E (31)	2022	Rat	Electrons	8 Gy/3fx	70-90 Gy/s	2	4	NA	>5.6 × 10^5^	No
						12.5 Gy/3fx	70-90 Gy/s	2.08	6	NA	>5.6 × 105	No
						15 Gy/3fx	70-90 Gy/s	1.85	6	NA	>5.6 × 10^5^	No
Total/ partial body	*In vivo*	Smyth LML (32)	2018	Mice	Photons	Totalbody:3.6-9.0	39.1	NA	NA	NA	NA	No
						Abdomen: 5.5-13.8	38.3	NA	NA	NA	NA	No
						Head: 7.6-18.9	41.3	NA	NA	NA	NA	No
						Toracic: 13.9-27.9	36.8	NA	NA	NA	NA	No

*Compared with conventional dose rate radiotherapy.

## 2 Dose rate

### 2.1 Brain

**Figure 1:**
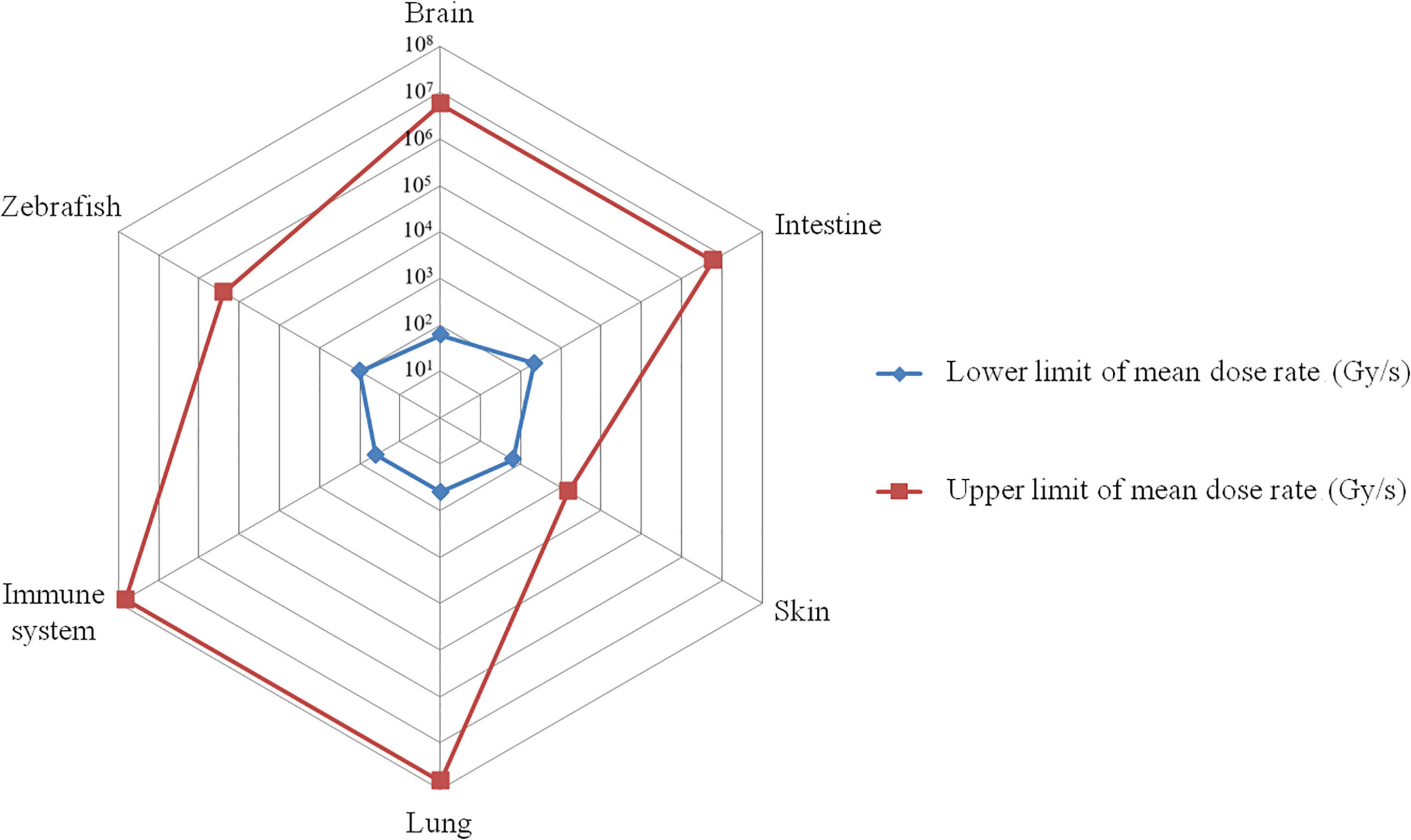
The mean dose rate range that triggers the FLASH effect. The red curve represents the highest mean dose rate reported in published research, while the blue curve represents the lowest mean dose rate. On the corresponding tissues, the FLASH effect appears between the blue and red curves (e.g. for brain, the dose rate range that triggers FLASH effect is between 10^1.78^~10^6.75^ Gy/s).

In 2017, Montay-Gruel et al. (14) delivered a uniform dose (10 Gy, electrons) to the mouse brain at various dose rates ranging from 0.1 Gy/s to 500 Gy/s and showed that the neuroprotective effect of FLASH-RT were diminished below 30 Gy/s but fully preserved above 100 Gy/s. Subsequently, their 2019 study confirmed that mean dose rates of ≥100 Gy/s (electrons) could produce FLASH effects in brain tissues (15). In 2021, they verified the neuroprotective and anti-tumor effects of FLASH-RT across different dose rates and fractionation regimens using electron beams (8). Mice with *in situ* glioblastoma received whole-brain irradiation with a single dose of either 10 Gy (mean dose rate 5.6×10^6^ Gy/s) or 14 Gy (mean dose rate 7.8×10^6^ Gy/s). While 10 Gy irradiation produced comparable but limited anti-tumor and neuroprotective effects in both groups, 14 Gy irradiation failed to exhibit neuroprotective effects. Subsequently, whole-brain irradiation with fractionated doses of 14 Gy/2F (mean dose rate 3.9×10^6^) and 30 Gy/3F (mean dose rate 5.6×10^6^) showed that FLASH-RT and CONV-RT were equally effective in delaying glioma growth; however, only FLASH-RT demonstrated a significant neuroprotective effect. Moreover, increasing the FLASH-RT dose further increased the suppression of tumor growth. FLASH-RT with an instantaneous dose rate exceeding 1.8×10^6^ Gy/s could effectively protect normal brain tissue from radiation-induced toxicity without compromising anti-tumor efficacy. In 2020, Allen et al. (16) exposed mice brains to electron beams delivering radiation doses of 10 Gy (mean dose rate 5.6×10^6^Gy/s) and 25 Gy (mean dose rate 2500 Gy/s), as well as an equivalent dose of CONV-RT (0.09 Gy/s). FLASH-RT reduced apoptosis level in neurogenic brain regions and induced less vasodilatation, thereby mediating a protective effect on the cerebral vasculature. In summary, a mean dose rate of 60 Gy/s or higher is required to elicit the FLASH effect in brain tissue (**Figure 1**). However, the mean dose rate is not the only condition impacting the FLASH effect. While dose partitioning has also been implicated as an influencing factor, the exact mechanism underlying the FLASH effect remains unclear and warrants further study (16).

### 2.2 Intestine

In 2021, Ruan JL et al. (17) investigated the impact of FLASH-RT on acute intestinal toxicity after whole-abdominal radiotherapy in mice. The mice were initially irradiated with electron beams delivering 7.5–12.5 Gy (mean dose rate 2.2–5.9×10^6^Gy/s), all of which produced FLASH effects on the intestine. Subsequent doses of 11.2 Gy and 12.5 Gy were delivered to the abdomen of mice at different mean dose rates (≥280 Gy/s or <280 Gy/s), revealing that FLASH effects could only be achieved at mean dose rates ≥280 Gy/s, which was also essential for minimizing intestinal crypts damage and maintaining intestinal microbiota integrity. In 2023, Zhang Q et al. (19) delivered various proton beam doses (14–18 Gy) to the abdomen of mice at a mean dose rate of 120 Gy/s, which failed to produce FLASH effects in the intestinal tissues, which may confirm the need for a mean dose rate exceeding 280 Gy/s.

However, a 2022 study by Zhu H et al. (18), delivering 10 Gy and 15 Gy X-ray doses to the abdomen of mice at a mean dose rate >150 Gy/s (instantaneous dose rate >5.5×10^5^Gy/s), showed improved survival and reduced intestinal damage in both groups compared to conventional radiotherapy, suggesting that mean dose rates exceeding 150 Gy/s can also produce the FLASH effect in intestinal tissues. In a 2020 study, Levy et al. (9) delivered electron beam doses of 12 Gy, 14 Gy, and 16 Gy at a mean dose rate of 216 Gy/s (instantaneous dose rate 4×10^5^ Gy/s) to irradiate the abdomen of mice. Remarkably, all doses within each group reduced the mortality rate of gastrointestinal syndromes and preserved intestinal function and epithelial integrity compared to conventional radiotherapy. Thus, a mean dose rate of 216 Gy/s can also produce FLASH effects on intestinal tissues (**Figure 1**). However, Zhu H et al.’s study reported an instantaneous dose rate exceeding 5.5×10^5^Gy/s despite the low mean dose rate, indicating the potential relevance of instantaneous dose rates in FLASH effect generation. Furthermore, proton, photon, and electron beams have varying beam pulse structures, and their instantaneous dose rates can differ by several orders of magnitude, which may affect the FLASH effect. However, the precise relationship between the instantaneous dose rate and FLASH effect occurrence remains unclear and requires further study.

### 2.3 Lungs

In 2014, Favaudon et al.’s study (2) showed that administering a ≥15 Gy dose of electron beam to the lungs of mice at a mean dose rate of ≥40 Gy/s (instantaneous dose rate 10^6^–10^7^ Gy/s) did not induce acute pneumonitis and pulmonary fibrosis and achieved comparable tumor control rates as CONV-RT. Moreover, increasing FLASH-RT doses were found to lead to complete tumor eradication. In 2019, a study showed that a mean dose rate of 60 Gy/s minimized the expression of pro-inflammatory genes in mouse lungs at an early stage, and reduced progenitor cell proliferation after radiotherapy, resulting in less DNA damage and senescent cells at later stages in the lungs. This suggests that FLASH-RT has a higher potential for lung regeneration, reduces DNA damage in normal cells, mitigates excessive damage to lung progenitor cells, and reduces the risk of replicative senescence (29). A mean dose rate of ≥40 Gy/s appeared to be the threshold for inducing FLASH effects in mouse lung tissue (**Figure 1**). However, other factors influencing this effect remain unclear and require further investigation.

### 2.4 Skin

Upon literature review, we found that the lowest mean dose rate that produces the FLASH effect in skin is 65 Gy/s (22) (**Figure 1**). However, a study in 2022 using a dose of 30 Gy (mean dose rate 1500 Gy/s, electrons) to irradiate nasal squamous cell carcinoma in cats and 31 Gy (mean dose rate 163 Gy/s, electrons) to irradiate the legs and shoulders of minipigs failed to elicit a FLASH effect in either group. In fact, the FLASH-RT group exhibited worse efficacy and more adverse effects (24). Despite the mean dose rate in this study surpassing the minimum threshold, the absence of a FLASH effect may be attributed to the excessively high single split dose exceeding the tolerance of the tissue. Therefore, in addition to the mean dose rate, factors such as single dose magnitude, tissue specificity, and instrument differences may influence FLASH effect development in skin.

### 2.5 Other systems

In 2020, Jin JY et al. (26) investigated the effect of FLASH-RT on circulating immune cells using computational modeling. A linear-quadratic model was fitted based on key parameters such as cardiac output, total blood volume, the dose delivered to the irradiated volume, the delivery time, and the blood circulation time for one cycle, to calculate the percentage of killed immune cells in circulating blood. Additionally, studies on the effects of ultra-high dose rates under various parameters were conducted. The results showed that within a dose interval of 2–50 Gy, when the mean dose rate was ≥40 Gy/s, the ultra-high dose rate had a strong protective effect on circulating blood cells, whereas a mean dose rate <40 Gy/s failed to induce a FLASH effect on circulating immune cells. In 2023, Cuitiño et al. (30) investigated the impact of FLASH-RT versus CONV-RT on the gonads of mice. Female mice were irradiated with 8 Gy (mean dose rate 2.33×10^6^ Gy/s, instantaneous dose rate 2.33×10^6^ Gy/s, electrons) or 16 Gy (mean dose rate 234 Gy/s, instantaneous dose rate 1×10^6^ Gy/s, electrons) to the abdomen, while male mice were irradiated with 5 Gy (mean dose rate 2.35×10^6^ Gy/s, instantaneous dose rate 2.34×10^6^ Gy/s, electrons) to the pelvis. No FLASH effects were observed in the gonadal organs (uterus, ovaries, and testes) of the female or male mice at all three doses. Although gonadal cells and lymphocytes are among the most radiosensitive cells in the human body, when irradiated with an average dose rate of ≥40 Gy/s, FLASH effects occurred in lymphocytes, but not in gonadal tissues. This underscores the influence of dose, instantaneous dose rate, and physical parameter differences among irradiating instruments on the FLASH effect.

## 3 Total dose

### 3.1 Brain

**Figure 2:**
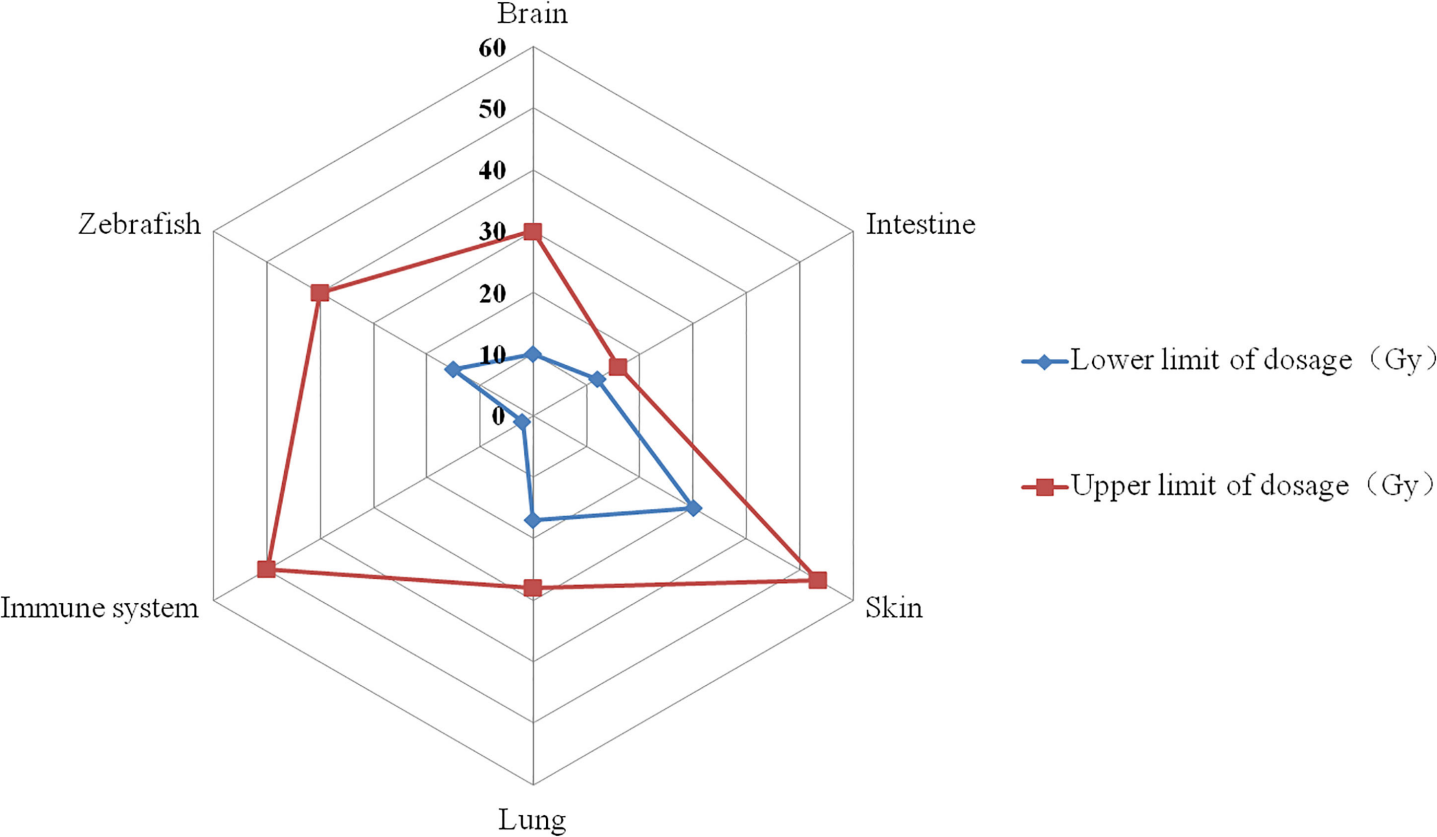
The dosage range that triggers the FLASH effect. The red curve represents the highest dose reported in published research, while the blue curve represents the lowest dose. On the corresponding tissues, the FLASH effect appears between the blue and red curves (e.g. for brain, the dosage range that triggers FLASH effect is between 10~30 Gy).

Several studies have assessed radiation-induced toxicity in the brain following CONV-RT and FLASH-RT in mice models. Montay-Gruel et al. (14) first found that a dose of 10 Gy electrons delivered via FLASH-RT (>100 Gy/s) preserved spatial memory, whereas CONV-RT (0.1 Gy/s) resulted in complete spatial memory impairment. This phenomenon was also confirmed after X-ray FLASH irradiation, which showed that a dose of 10 Gy did not cause memory deficits, reduced hippocampal cytokinesis damage, or diminished reactive astrogliosis (33). In addition, FLASH-RT did not induce anxiety or depressive behaviors at six months post-irradiation. One month post-irradiation, a series of spontaneous exploratory tasks in mice revealed no performance decline in the 12 Gy group, although the FLASH effect eventually waned in the 14 Gy exposure cohort (15). A dose-fractionation approach was used to validate the neuroprotective effects of FLASH-RT using electron beams. Notably, only the 10 Gy/1F, 10 Gy/2F, and 30 Gy/3F groups exhibited no neurocognitive decline, whereas the 14 Gy/1F, 14 Gy/4F, and 25 Gy/1F groups exhibited no neuroprotective effect. Therefore, neurocognitive sparing can be achieved through FLASH-fractionated regimens (8). Another study reported that FLASH-RT using electron beams reduced the apoptosis level in neurogenic regions and maintained microvascular system integrity in the brain when administered to C57Bl/6J female mice at a single dose of 25 Gy (>106 Gy/s) and 10 Gy (>106 Gy/s), as observed at both the early (24 h, 1 week) and late (1 month) post-irradiation endpoints (16). Furthermore, FLASH-RT (30 Gy/1 F, 300 Gy/s, and 200 Gy/s, electrons) reduced neuroinflammation induction, whereas CONV-RT led to a significant increase in five out of ten pro-inflammatory cytokines in the hippocampus (34). Smyth et al. (32) used a total dose of 7.6-18.9 Gy/1F (41.3 Gy/s, photons) to irradiate the heads of mice and found that the FLASH effect disappeared. Based on these results, we can infer that the neuroprotective effects of FLASH are primarily observed within the 10–30 Gy dose range (**Figure 2**) and that this effect may be altered by fractionation approaches.

### 3.2 Intestine

Numerous researchers have explored the FLASH effect of abdominal irradiation using various radiation sources, such as electrons, protons, and high-energy X-rays. Upon comparing whole-abdominal irradiation in normal and ovarian cancer mice using FLASH-RT (12–16 Gy, 216 Gy/s, electrons) and CONV-RT (12–16 Gy, 0.079 Gy/s, electrons), Levy et al. (9) found that 14 Gy irradiation induced less apoptosis and early DNA damage in crypt basal columnar cells, resulting in better preservation of intestinal function and epithelial integrity. Ruan JL et al. (17) investigated the effect of different temporal pulses and dose rates using electron beams on intestinal effects in mice and found that gastrointestinal function was preserved at doses between 7.5–12.5 Gy (2.2~5.9×10^6^ Gy/s), 11.2 or 12.5 Gy/1F (<280 Gy/s), but not at 11.2 or 12.5 Gy/1F (≥280 Gy/s). Interestingly, the diversity of the gut microbiota was also differentially affected by FLASH irradiation.

The sparing effect of high-energy X-ray FLASH-RT (12 Gy/1F, 700 Gy/s, 6 MeV) and CONV-RT (12 Gy/1F, 0.1 Gy/s, 6 MeV) was first compared in 2019. The survival time of healthy mice irradiated in the abdomen was higher in the FLASH group (12–15 Gy) than that of the CONV-RT group (7 days), with 62.5% of mice still alive at the end of observation (11). In 2022, the FLASH-RT group (12–15 Gy) of mice irradiated with the same radiation device experienced less acute intestinal injury than the CONV-RT group (18).

Whole-abdominal irradiation at 15 Gy significantly reduced the proliferation rate of each crypt cell, with proton FLASH-RT sparing more than CONV-RT (35). However, partial abdominal FLASH-RT irradiation (14-18 Gy, 120 Gy/s, 228 MeV, proton) delivered to C57BL/6J and immunodeficient Rag1-/-/C57 mice did not spare normal intestinal tissue, and no difference in lymphocyte depletion was observed (19). Hence, FLASH-RT efficacy may depend on various factors, and dose rates above 100 Gy/s may not induce a FLASH effect or may yield worse outcomes.

Overall, the dose range for the FLASH-RT intestinal-sparing effect tends to be between 12–16 Gy (**Figure 2**), a relatively low value considering that gastrointestinal cells are among the most radiosensitive cells in normal tissues. Thus, the FLASH effect may be influenced by different pulses, radiation sources, and dose rate factors.

### 3.3 Lungs

C57BL/6 mice were exposed to FLASH-RT (17–28 Gy, ≥40 Gy/s, 4.5 Mev, electron) or CONV-RT (<0.03 Gy/s) through bilateral thorax irradiation to investigate radiation-induced toxicity. The study revealed that 100% of mice in the CONV-RT group developed severe pneumonia and pulmonary fibrosis, whereas FLASH-RT inhibited the activation of the TGF-β/SMAD cascade response, protected the blood vessels and bronchioles from radiation-induced apoptosis, and reduced the incidence of pulmonary fibrosis (2). In another study using C57BL/6J wild-type and Terc-/- mice, human lung cells cultured *in vitro* were subjected to bilateral thorax irradiation using electron beams, followed by quantitative PCR and single-cell RNA sequencing (sc-RNA-Seq). Histological methods were employed to examine lung responses to FLASH-RT (*in vivo*: 17 Gy; *in vitro*: 2–5 Gy). FLASH-RT was found to reduce DNA damage in normal cells, spare lung progenitor cells from excessive damage, and decrease the risk of replicative senescence (29). Interestingly, in an experiment using mouse lung carcinomas, tumors treated with proton FLASH-RT (18 Gy) were significantly smaller than those irradiated with CONV-RT. Moreover, proton FLASH-RT was found to increase CD3+ T lymphocyte recruitment from the tumor margins to the tumor core, as well as increase in CD4+ and CD8+ cells, potentially contributing to the high tumor control observed in the FLASH-RT group (36). These studies collectively suggest that a dose range of 17–28 Gy (**Figure 2**) for thoracic FLASH-RT to confer lung-protective effects. However, given the limited sample sizes in those studies, more basic experiments are needed in the future.

### 3.4 Skin

Since the discovery of FLASH effects, the skin system has been extensively studied, including validation in mammalian models and human patients. FLASH-RT demonstrated a significant normal tissue-sparing effect in a study involving right hind limb irradiation in CDF1 mice using CONV-RT or FLASH-RT (31.2–53.5 Gy/1F, 65–92 Gy/s, protons), with the observation endpoint being the level of acute moist desquamation of the foot skin 25 days post-irradiation (22). Similarly another proton FLASH-RT study (30 Gy/1F or 45 Gy/1F, 69–125 Gy/s) reported a reduction in skin damage, stem cell depletion, and inflammation (21). In the first report of electron FLASH-RT on skin toxicity, a single dose range of 10-40 Gy was delivered to mouse skin. The FLASH effect occurred at relatively high doses (30/1F and 40 Gy/1F), while no significant protective effect was observed in the lower-dose groups (10 Gy/1F, 16 Gy/1F, and 20 Gy/1F) (20). Konradsson et al. (31) also observed no significant differences in overall survival, acute side effects, or late side effects in Fisher 344 rats irradiated with electron FLASH-RT(8 Gy/3F, 12.5 Gy/3F, 15 Gy/3F, and ≥70 Gy/s). In healthy FVB/N and FVBN/C57BL/6 outbred mice irradiated on one hind leg using FLASH (35 Gy, 43 Gy, 87 Gy/s, X-rays) and CONV-RT (<0.05 Gy/s), histopathological assessment revealed potential radioprotective effects in the 35 Gy group at 8-weeks post-irradiation. However, no significant differences were observed between FLASH-RT and CONV-RT samples at 43 Gy for the less-severe endpoints used for histopathological assessment (23).

A phase I clinical trial studied six cats with locally advanced nasal plane T2/T3N0M0 squamous cell carcinoma, with acute and late observation endpoints of alopecia and fibrotic necrosis, respectively. No acute toxicity was observed in three cats in the FLASH-RT group (25–41 Gy, 4.5 or 6 MeV, electron), while the remaining three cats developed moderate/mild transient mucositis. A 16-month progression-free survival rate of 84% was reported. In mini-pigs who received electron beams CONV-RT (≈5 Gy/min) and FLASH-RT (22–34 Gy, ≈300 Gy/s), one developed skin patches three weeks after exposure to FLASH-RT, however this was transient (lasting only four weeks) for doses ≤31 Gy. Conversely, the hair follicles of those exposed to CONV-RT appeared to be permanently destroyed, with no hair regrowth observed 6 months post-irradiation. This study confirmed the potential benefits of FLASH-RT (6). However, osteoradionecrosis developed in 3 out of 7 cats who received FLASH-RT (30 Gy) at a mean dose rate of 1500 Gy/s, resulting in preliminary termination of the trial (24). In this study, mini pigs were irradiated using applicators of increasing size and a single surface dose of 31 Gy FLASH-RT. Although no acute toxicity was recorded, severe late skin occurred in a volume-dependent manner (7–9 months). This suggests that investigators should be aware of the total dose and dose rate of FLASH-RT and emphasizes the need for further investigation.

In Switzerland, a patient with cutaneous lymphoma was treated on the same day for two distinct tumors using FLASH-RT (15 Gy, 166 Gy/s, electron) and CONV-RT (15 Gy, 0.08 Gy/s). However, there was no difference in acute reactions, late effects at 2 years, and tumor control (25). Thus, the dose range for the FLASH skin-sparing effect tends to be between 30–53.5 Gy (**Figure 2**). It is important to note that assessing skin reactions requires attention to the time of observation.

### 3.5 Other systems

Few studies have evaluated the effects of FLASH-RT on the gonadal and immune systems. Using a linear accelerator, the abdomen or pelvis of female (8 Gy, 2.31×10^6^ Gy/s or 16 Gy, 234 Gy/s) and male (5 Gy, 2.31×10^6^ Gy/s) C57BL/6J mice, respectively, were irradiated with electron beams. The ovaries of both CONV-RT and FLASH-RT mice exhibited similar follicular deficiencies, while the weight of the testes was reduced, and the percentage of degenerate seminiferous tubules was much higher than that of the controls (30).

Moreover, Venkatesulu et al. (37) reported that a cumulative dose of 5–10 Gy (35 Gy/s, 20MeV, electrons) did not protect mice from the deleterious side effects of radiation in a cardiac and splenic radiation-induced lymphocyte reduction model following mucosal injury. Hence, lymphocyte sensitivity to radiation, a key driver of adverse radiation effects, may not fully explain the wide therapeutic window of FLASH-RT.

Interestingly, one computation-based study calculated a strong sparing effect on circulating immune cells by FLASH-RT and reported that this sparing effect increased with dose/fraction, reaching a plateau at 30–50 Gy/1F and almost completely vanishing at 2 Gy/1F. This may contribute to the reported FLASH effects in animal studies (26).

## 4 Pulse structure

Since the dose rate and total dose cannot completely determine FLASH generation (27), some researchers believe that the pulse structure may be an important factor affecting the FLASH effect (38). The dose delivery of FLASH-RT typically consists of a single or multiple pulses, with the characteristic parameters of the pulse structure including single-pulse dosage (instantaneous dose rate), pulse width, pulse interval, pulse frequency, and total irradiation time (39).

In 2019, Bourhis et al. (40) summarized previous studies on FLASH-RT brain injury and inferred that the total irradiation time, single-pulse dosage, and number of pulses may have an impact on the FLASH effect. In response, Montay-Gruel et al. (8) concluded that FLASH-RT needs to achieve a single-pulse dosage of 10^5^–10^6^ Gy/s or higher to confer a protective effect on brain cognitive function. Ruan et al. (17) fixed the total dose at 11.2 Gy and explored the severity of intestinal injury in mice exposed to electron FLASH-RT with different pulse counts (1, 2, 5, 30, 100, and 300) and pulse intervals (3.3×10^-3^ s, 0.010 s, 0.040 s, 3 s, and 30 s), and found that as the number of pulses increased or the pulse interval was prolonged, the number of intestinal crypts decreased. Given the direct impact of the number and interval of pulses on the average dose rate of FLASH-RT, the authors proposed that the pulse structure could influence the FLASH effect by modulating the average dose rate, whereby a higher average dose rate was associated with a more potent protective effect of FLASH-RT on the intestine.

Recently, Karsch et al. (28) published an *in vivo* study on the effects of pulse structures on the FLASH effect using electron beams, whereby zebrafish were divided into four groups (30 per group): the reference (CONV-RT group, total dose: 31.5 ± 0.6 Gy, average dose rate: 0.12 Gy/s, irradiation time: 240 s, instantaneous dose rate: 1.8 Gy×10^3^ Gy/s), UHDRiso (the pulse structure of a clinical iso chronous cyclotron, total dose: 31.9 ± 0.5 Gy, average dose rate: 286.7 Gy/s, irradiation time: 0.1 s, instantaneous dose rate: 4.4×10^6^ Gy/s), UHDRsynchro (the pulse structure of a clinical synchrocyclotron, total dose: 32.3 ± 0.6 Gy, average dose rate: 177.2 Gy/s, irradiation time: 0.164 s, instantaneous dose rate: 1.4×10^9^ Gy/s), and UHDRmax (the maximal available pulse dose rate of used accelerator, total dose: 32.1 ± 0.6 Gy, average dose rate: 2.5 ×10^5^ Gy/s, irradiation time: 0.0003 s, instantaneous dose rate: 1.5×10^9^ Gy/s) groups. The pulse width and frequency used in the four groups were 5 ps and 13 MHz, respectively. The main difference between the UHDRiso group and UHDRsynchro group was the instantaneous dose rate (4.4×10^6^ Gy/s vs 1.4×10^9^ Gy/s). By incorporating macro pulses (each consisting of 800 pulses at a frequency of 20 Hz and width of 40 ms) to the UHDRsynchro group, the average dose rates of the UHDRiso and UHDRsynchro groups were comparable, with no order of magnitude difference (286.7 Gy/s vs 177.2 Gy/s). The UHDRmax group and UHDRsynchro also exhibited similar instantaneous dose rates, but due to the absence of a macro pulse structure, the average dose rate of the UHDRmax group was much higher than that of UHDRsynchro (2.5×10^5^ Gy/s vs 177.2 Gy/s). The UHDRmax group also conferred the best protective effect on zebrafish, while the UHDRiso and UHDRsynchro groups conferred similar protective effects, and the reference group exhibited the most severe damage. Karsch et al. also conducted a proton FLASH-RT (average dose rate of 300 Gy/s, instantaneous dose rate of 1.5×10^3^ Gy/s) study, where the protective effect of proton FLASH-RT on zebrafish was similar to that of the UHDRiso group and UHDRsynchro group, but worse than that of the UHDRmax group. The authors proposed that the strength of the FLASH effect was positively correlated with the average dose rate and that the instantaneous dose rate and radiation source may not be influencing factors of the FLASH effect.

While the studies by Ruan et al. and Karsch et al. have confirmed that pulse structures may affect the FLASH effect, several issues that warrant further exploration remain. First, due to the direct impact of changes in pulse characteristic parameters on the average dose rate, current research on the impact of pulse structure parameters on the FLASH effect tends to attribute changes to the average dose rate. Therefore, more systematic radiobiological research on different pulse structures is needed, especially by adjusting the pulse structure while maintaining the same average dose rate and total dose. Second, owing to the difficulty in modulating pulse structures on the same proton device, no research on the FLASH effect of pulse structures on protons has been conducted to date. Instead, research on pulse structures has primarily focused on electrons (17, 28) and X-rays (10). Unlike electron beams, the number of pulses appear to have no effect on the generation of X-ray FLASH effects (10). Thus, further research is needed to determine whether the generation of FLASH effects with different types of radiation is related to pulse structure.

## 5 Radiation sources

At present, the radiation sources used in FLASH radiotherapy research include electrons, protons, photons, etc. (3). The implementation of ultra-high dose rate electron beams present relatively minimal difficulty. However, given the limited penetration capability, electron beams are primarily suited for the treatment of superficial tumors. Proton beams may be suitable for deep tumors, but the high construction and operating costs limits its application. High-energy X-rays penetrate deeply, have a small divergence, and are affordable. However, due to the difficulty in implementing ultra-high dose rate high-energy X-rays, currently only our team has established a stable experimental platform globally (PARTER, 6-8 MeV, ~1000 Gy/s) (11). Recently, Thomas W et al. compared the oxygen consumption and reactive oxygen species production in water following protons and electrons FLASH radiotherapy. They found that electron beams FLASH radiotherapy consumed more oxygen and produce less H_2_O_2_ (41). Moreover, Liu K et al. utilized synchrotron-based proton and linac-based electron beams for FLASH irradiation to investigate the protective effects of different radiation beams on the acute gastrointestinal toxicity of mice. They found that electron beams resulted in higher survival rates and greater gut crypt numbers (42). However, in the comparative study by Kacem H et al. on the protective effects of electron beams and proton beams FLASH radiotherapy, the protective effects of the two types of radiation beams on zebrafish were consistent (43). Overall, there are relatively few studies that directly compare the biological effects of different types of radiation beams, and there is currently a lack of research comparing photons with other types of radiation sources.

## 6 Conclusion

This study provides an overview of the beam parameters influencing the FLASH effect and proposes that an average dose rate of 40 Gy/s appears to be the lowest dose that triggers this effect. Beyond this threshold, different organs required varying minimum single total doses to trigger FLASH effects, with a trend of enhanced FLASH-RT protective effects as the single total doses increased. Moreover, different radiation sources, single or multiple pulses and the characteristic parameters of the pulse structure, including single pulse dosage, pulse width, pulse interval, pulse frequency, and total irradiation time, may also impact the FLASH effect. However, current limitations in FLASH-RT equipment hinder comprehensive research on pulse structures. In addition, it should be noted that the uncertainty in doses in some studies can be on the order of 5% or worse, and this uncertainty in doses may also impact the reproducibility of the results. The interaction between dose rate, total single dose, and pulse structure on the FLASH effect remains complex and poorly understood, necessitating further research.
